# Design and Analysis of a Biodegradable Polycaprolactone Flow Diverting Stent for Brain Aneurysms

**DOI:** 10.3390/bioengineering8110183

**Published:** 2021-11-12

**Authors:** Kaitlyn Tidwell, Seth Harriet, Vishal Barot, Andrew Bauer, Melville B. Vaughan, Mohammad R. Hossan

**Affiliations:** 1 Department of Engineering and Physics, University of Central Oklahoma, Edmond, OK 73034, USA; ktidwell4@uco.edu (K.T.); sharriet@uco.edu (S.H.); vbarot@uco.edu (V.B.); 2 Department of Neurosurgery, University of Oklahoma-Health Science Center, Oklahoma City, OK 73104, USA; andrew-bauer@ouhsc.edu; 3 Department of Biology, University of Central Oklahoma, Edmond, OK 73034, USA; mvaughan4@uco.edu; 4 Center of Interdisciplinary Biomedical Education and Research, University of Central Oklahoma, Edmond, OK 73034, USA

**Keywords:** flow diverting stents (FDS), biodegradable, aneurysms, Polycaprolactone (PCL) flow diverters, endovascular treatment

## Abstract

The flow diverting stent (FDS) has become a promising endovascular device for the treatment of aneurysms. This research presents a novel biodegradable and non-braided Polycaprolactone (PCL) FDS. The PCL FDS was designed and developed using an in-house fabrication unit and coated on two ends with BaSO_4_ for angiographic visibility. The mechanical flexibility and quality of FDS surfaces were examined with the UniVert testing machine, scanning electron microscope (SEM), and 3D profilometer. Human umbilical vein endothelial cell (HUVEC) adhesion, proliferation, and cell morphology studies on PCL FDS were performed. The cytotoxicity and NO production by HUVECs with PCL FDS were also conducted. The longitudinal tensile, radial, and bending flexibility were found to be 1.20 ± 0.19 N/mm, 0.56 ± 0.11 N/mm, and 0.34 ± 0.03 N/mm, respectively. The FDS was returned to the original shape and diameter after repeated compression and bending without compromising mechanical integrity. Results also showed that the proliferation and adhesion of HUVECs on the FDS surface increased over time compared to control without FDS. Lactate dehydrogenase (LDH) release and NO production showed that PCL FDS were non-toxic and satisfactory. Cell morphology studies showed that HUVECs were elongated to cover the FD surface and developed an endothelial monolayer. This study is a step forward toward the development and clinical use of biodegradable flow diverting stents for endovascular treatment of the aneurysm.

## 1. Introduction

The flow diverting stent (FDS) was first approved in 2011 by the US Food and Drug Administration (FDA) for treatment of large or wide-necked aneurysms of the internal carotid artery [1]. Later, the application of FDS was expanded for the treatment of small and medium wide-necked aneurysms [1]. The FDS promotes aneurysm occlusion using three distinct hemodynamic mechanisms: decreasing direct jet blood flow into the aneurysm, promoting laminar flow along the direction of the artery, and decreasing the speed of the blood flow in the aneurysm [2]. The impact of these hemodynamic changes causes intra-aneurysmal thrombosis and subsequent aneurysmal occlusion with complete occlusion reported in 76–94.2% of patients in the time span between six months to five years of post-operation [3,4,5,6]. However, post-treatment complications such as neointimal hyperplasia, ischemic or thromboembolic events, hydrocephalus, hemorrhagic events, aneurysm rupture, incomplete occlusion and parent artery occlusion, among others, are also reported in various studies [4,7,8,9]. Thrombotic and stenotic events were also observed with coronary stents during cardiovascular intervention [10,11]. Metallic stents cause mechanical stress on vascular walls and low-grade injury due to micromotion, dislodgement, and mispositioning [12,13]. Thus, it may become a source of persistent mild inflammation, subacute thrombosis and in-stent restenosis for an indefinite period of time [14]. The permanent placement of metallic stents limits reactive vasomotor functions, revascularization, and may interfere with non-invasive assessment such as MRI and X-rays [15,16,17]. Hence, biodegradable stents (BDS) are proposed to resolve many of these challenges [18].

The BDS provides scaffolding support to the damaged vessel, reduces the risk of late stent thrombosis and prevents elastic recoil and constrictive remodeling [19]. While BDSs can greatly improve patient outcomes, the following challenges still need to be resolved before widespread clinical applications are possible [18]. Stents lack bending flexibility to follow the wavy curvature of blood vessels without injuring the vessel. Similarly, they lack enduring radial strength to promote long-term healing [20,21]. The immature and nonuniform degradation rates of the BDS compromise the mechanical integrity of the BDS and cause vascular inflammatory responses [14]. Other challenges include appropriate thickness, strut designs, biocompatibility, and selection of BDS materials [14,22,23]. However, efforts are ongoing to resolve the above-mentioned challenges through various experimental studies and computational modeling for appropriate mechanical properties [24,25], degradation rates [26,27], BDS designs and material development [28].

Among various biomaterials for biodegradable stents, PCL has unique potential due to its biocompatibility, flexibility, and the user’s ability to fine-tune the mechanical properties of PCL using composites or specific processing techniques [29,30,31,32]. The melting point and glass transition-point temperature of PCL are around 60 °C and −60 °C [33], respectively. These make it a suitable candidate material for biodegradable implants in both hard and soft tissues [34,35]. The flexibility, degradation rate, drug release rate, dyeability, adhesiveness, and stress-crack resistance of PCL are reportedly enhanced by blending PCL with other materials like cellulose acetate butyrate, cellulose propionate, polylactic acid-co-glycolic acid, β-tricalcium phosphate (β-TCP), among other materials [31,36,37]. Studies of PCL have revealed that chondrocytes, osteoblasts, fibroblasts, nerve cells, muscle cells, and endothelial cells all favorably adhere to and proliferate on PCL scaffolds [38,39,40]. Recently, a handful of investigations on PCL-based coronary stents was also reported in the literature [41,42].

Despite various reports on the in vitro and in vivo studies of biodegradable stents and scaffolds for interventional cardiovascular applications, there are only a handful of studies on the biodegradable flow-diverting stents for the endovascular treatment of aneurysms. While the long-term goal of our research is to develop an efficient biodegradable flow-diverting stent for clinical applications of brain aneurysms, here we present a novel, non-braided PCL-based biodegradable FDS with mechanical and in vitro biocompatibility analysis. PCL has already been approved for use in drug delivery devices and shows excellent biocompatibility both initially and throughout the degradation process [29,43]. The two ends of FDS were coated with BaSO_4_ for angiographic visibility during the deployment. Bending, radial, and longitudinal strength and flexibility were evaluated via mechanical analysis, while cell response analysis was performed using human umbilical vein endothelial cells (HUVECs). The results are promising and comparable to the commercial coronary stents. This study is a step forward toward the development of an efficient, biodegradable flow-diverting stent for brain aneurysm treatment.

## 2. Materials and Methods

### 2.1. Chemicals

All chemicals were purchased from Sigma-Aldrich, Inc. (St. Louis, MO, USA) if not mentioned otherwise. Medical grade PCL filament was purchased from Advanced Biomedical Technology Inc. (Hsinchu City, Taiwan). The Nitrate/Nitrite Colorimetric Assay Kit was purchased from Cayman Chemical (Ann Arbor, MI, USA). The CyQUANT™ LDH Cytotoxicity Assay Kit was purchased from Thermo Fisher Scientific (Waltham, MA, USA). The VascuLife^®^ Basal Medium and the VascuLife^®^ VEGF LifeFactors Kit were purchased from Lifeline Cell Technology (Frederick, MD, USA). The NucSpot^®^ 470 Nuclear Stain, 1000x in DMSO, was purchased from Biotium, Inc. (Fremont, CA, USA). The EdU-Click 594 Cell Proliferation Kit was purchased from baseclick GmbH (Munich, Germany). The lab-grade BaSO_4_ was purchased from Lab Alley (Spicewood, TX, USA). The acetone was purchased from Lab Chem Inc. (Zelienople, PA, USA). The hexamethyldisilazane (HMDS), as well as the glutaraldehyde, were purchased from Electron Microscopy Sciences (Hatfield, PA, USA). The phosphate buffered saline (PBS) was purchased from Life Technologies Limited (Paisley, PA, USA). The formaldehyde was purchased from Alfa Aesar Thermo Fisher Scientific (Ward Hill, MA, USA).

### 2.2. Design of Fabrication Unit and Flow Diverting Stent

**Figure 1:**
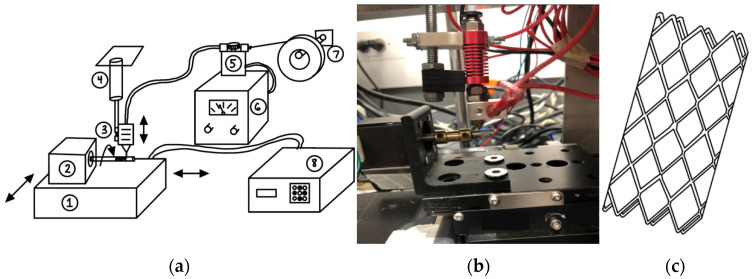
(**a**) Schematic of in-house fabrication unit of flow diverting stent (FDS) shows different parts of the unit as follows: Micro-motion stage with two degrees of freedom (1), rotary arm FDS forming platform (2), electromelt needle (3), vertical micromotion stage (4), polycaprolactone (PCL) feed rate controller (5), variable power supply (6), filament spool hanger (7) and micro-motion controller unit (8); (**b**) the actual fabrication unit and (**c**) schematic of the PCL FDS.

The flow diverting stent fabrication unit was developed based on a 3D micromotion stage (Newport Corporation, Franklin, MA, USA), variable diameter rotary arm, electromelt extruder, temperature controller and a cooling fan as shown in Figure 1. The micromotion stage can move in x, y and z directions with an increment of 1 micrometer. The rotational motion of the rotary arm was controlled by a nema-17 stepper motor with 200 steps per revolution (i.e., 1.8-degree turning precision). A Newport esp301 3-axis controlled the movement of the micromotion stage while the rotary motion was controlled by an Ardinuo Uno with microstep driver (LAFVIN, Shenzhen, China). Feed rate of the filament materials in the electromelt extruder was controlled with a separate feed gear with Ardinuo nano (LAFVIN, Shenzhen, China). The FDS was first conceptualized and analyzed using the CAD program as shown in Figure 1c. The relevant Python code was developed, tested, and optimized for micromotion stage movement, rotary arm rotation, and feed rate. The medical-grade PCL filament (Advanced Biomedical Technology Inc., Hsinchu City, Taiwan) was used to demonstrate this novel PCL FDS. The nominal diameter of the FDS varied based on the diameter of the rotary arm. The rotary arm was designed through CAD modeling and printed in a 3D printer (Sonic Mini 4K, Phrozen, Hsinchu City, Taiwan).

### 2.3. Surface Characterization and Mechanical Testing

PCL FDSs (15 mm long and 5.5 mm nominal diameter) were fabricated using our in-house fabrication unit. FDSs were cleaned with isopropyl alcohol (IPA) and DI water, dried, and cut into 5 mm by 2 mm pieces for surface characterization. Surfaces were examined under a scanning electron microscope (SEM, Hitachi TM3000 Tabletop, Tokyo Japan) and surface roughness was measured with the 3D profilometer (Profilm3D, Filmetrics, San Diego, CA, USA). The mechanical analysis of the PCL FDSs was conducted with three-point bending, radial compression, and longitudinal tensile tests based on ASTM standard procedures using the UniVert testing machine (CellScale, Waterloo, ON, Canada). Bending and flat plate radial tests were conducted using the UniVert testing machine manufacturer-provided fixtures, while a 3D printed fixture was used to conduct tensile tests. The FDS were held in the tensile test fixture through a pin. Similar testing fixtures were used for mechanical analysis of coronary stents in previous studies [44,45]. A 20 N load cell was used for the tests with a resolution of 0.001 N. The PCL FDS was compressed to about 0.6 mm between flat plates. The unloading rate for the bending and radial test was 0.211 mm/s and 0.15 mm/s, respectively. The bending moment and the bending flexibility were calculated using the following equations [44]:

(1) $$M=\frac{PL}{4}$$

(2) $$F_{b}=\frac{PL^{3}}{48\delta }$$

where *M* is the bending moment, *P* is the applied load, *L* is the length of the FDS between the end supports, *F_b_* is the bending flexibility, and *δ* is the deflection of the FDS at the center. The normalized radial strength was estimated using the following equation [44] to estimate the resistance of the FDS against radial deformation.

(3) $$F_{r}=\frac{P_{i}d}{\delta_{i}}$$

where *F_r_* is the normalized radial strength, *P_i_* is the applied force, *δ_i_* is the deformation corresponding to applied force, and *d* is the initial nominal diameter of the FDS.

### 2.4. BaSO_4_ Coating for X-ray Image Analysis

The two ends of the PCL FDS were coated with BaSO_4_ (Sigma-Aldrich, Inc., St. Louis, MO, USA) for the visibility of the FDS during deployment and post-treatment pathological evaluation under X-ray or angiographic imaging. The appropriate composition of coating was determined by testing four different W/V% solutions of BaSO_4_ and PCL in acetone. The solutions were made by mixing 15% BaSO_4_: 5% PCL, 20% BaSO_4_: 5% PCL, 20% BaSO_4_: 10% PCL, and 30% BaSO_4_: 10% PCL in 5 mL of acetone for 60 min using a sonicator probe (Sonics Vibracell, VC130, Sonics & Materials Inc, Newtown, CT, USA). Pre-washed, square PCL tiles (10 mm by 10 mm) were fully coated with the various compositions of BaSO_4_ and PCL solutions to determine the optimum coating. After drying completely, X-ray images were obtained by using a handheld X-ray machine (MaxRay DX-3000, Iridium Dental, University Place, WA, USA) with an exposure time of 1.35 s at a distance of 20 cm from the sample. The X-ray images were analyzed for the peak intensity using ImageJ software (National Institute of Health, Bethesda, MD, USA). These solutions were then used to coat the ends of PCL FDS samples, and X-ray images were obtained for analysis. The 30% BaSO_4_–10% PCL and 20% BaSO_4_–10% PCL coatings of PCL tile were also tested for the possible adverse effects (if any) on cell adhesion and proliferation by seeding 1 × 10^5^ cells/mL on flat PCL tiles in a 48-well plate. The details of the procedure are described in Section 2.6.

### 2.5. HUVEC Cytotoxicity and NO Production Studies

All cell culture was performed using standard aseptic techniques. HUVECs and VascuLife^®^ Basal Medium containing LifeFactors^®^ Vascular Endothelial Growth Factor kit (Lifeline^®^ Cell Technology, Frederick, MD, USA) were used for all cell studies. Cells were cultured in a humidified atmosphere at 37 °C with 5% CO_2_ until confluent, then washed with Phosphate Buffered Saline (PBS), lifted with 1x Trypsin, neutralized with trypsin neutralizing solution, and counted. The toxicity of the PCL FDS Samples was analyzed using the Invitrogen™ CyQUANT™ LDH Cytotoxicity colorimetric assay kit (Thermo Fisher Scientific, Waltham, Massachusetts, USA). The optimum cell number (75 cells/µL) was determined according to the manufacturer’s protocol. The LDH Cytotoxicity was found by plating the cells on sterilized, flat PCL FDS samples, as well as spontaneous, maximum, and media only control wells in a 24-well tissue culture plate. The cells were incubated for 24-, 36-, and 48-h at 37 °C with 5% CO_2_. After incubation, the assay was performed according to the manufacturer’s protocol. To determine the total NO production by HUVECs in response to the PCL FDS samples, 12,500 cells/well were plated on PCL FDS samples and a media-only control in a 24-well plate. The cells were allowed to incubate at 37 °C with 5% CO_2_ for 24-, 36-, and 48-h. At each respective time period, the total NO production was found using Cayman Chemical’s Nitrate/Nitrite Colorimetric Assay Kit (Cayman Chemicals, Ann Arbor, MI, USA) according to the manufacturer’s protocol.

### 2.6. Proliferation, Adhesion, and Cell Morphology Analysis

PCL FDS samples were washed, sterilized under UV light for 1 h, and then plated with 1 × 10^5^ HUVECs/well 24-well tissue culture plate with 1 mL of media. The samples were incubated at 37 °C with 5% CO_2_ for 24-, 36-, and 48-h. For each set of samples, after the initial incubation time, the samples were moved into a new well containing half old media, half new media, and 10 µM 5-ethynyl-2′-deoxyuridine (EdU). The samples were incubated for an additional 24 h before fixation with 4% paraformaldehyde, washing with 3% bovine serum albumin in PBS, and permeabilization with 0.5% Triton^®^ X-100 in PBS. After fixation, the samples were washed and stained using the BaseClick^®^ EdU-594 proliferation stain kit (BaseClick GmbH, Munich, Germany) according to the manufacturer’s protocol. Then, the samples were counterstained with NucSpot^®^ 470 green nuclear stain (Biotium Inc., Fremont, CA, USA) for 10 min, according to the manufacturer’s procedure. Finally, the samples were mounted onto a glass cover slide using 80/20 glycerol/PBS and analyzed with a fluorescent microscope (Olympus IX-71 inverted epifluorescent microscope, Olympus Corporation, Tokyo, Japan). The samples were photographed under a fluorescein isothiocyanate (FITC) filter as well as a Texas Red filter. For morphological analysis of the HUVECs plated on PCL FDS samples, samples were removed and fixed with 4% glutaraldehyde at each respective time period. Then the samples were washed three times with phosphate buffer, three times with deionized water, and then dehydrated in a graded ethanol series. Next, the samples were submerged in HMDS three times for 10 min each and allowed to air dry. Finally, the samples were mounted onto stubs, sputter-coated with a thin layer (~ 5 nm) of AuPd, and analyzed using SEM.

### 2.7. Statistical Analysis

All measurements and experiments were conducted at least 3 times, and data are presented as mean with plus or minus standard error of mean/standard deviation. The data were analyzed using Microsoft Excel (Microsoft, Seattle, WA, USA) by Student *t*-test and a *p*-value of <0.05 was considered statistically significant.

## 3. Results

### 3.1. Surface Characterization and Mechanical Testing

**Figure 2:**
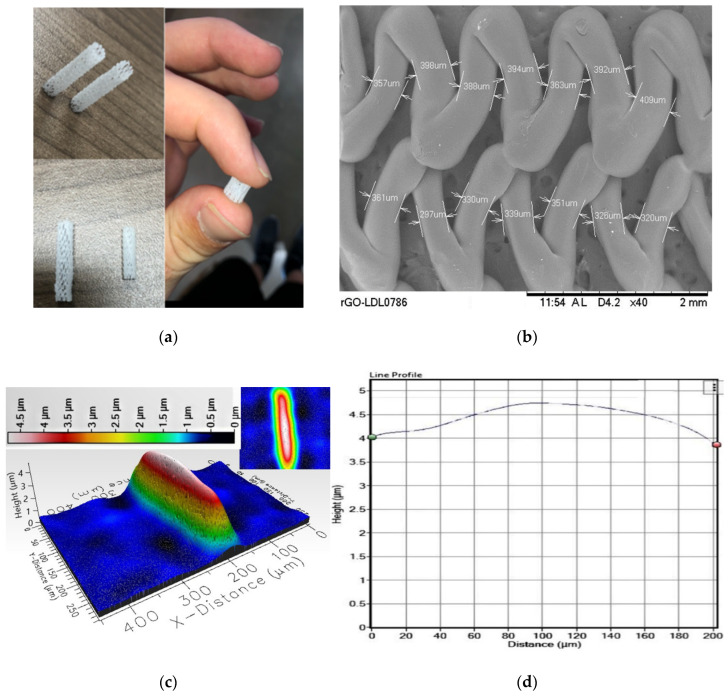
(**a**) Developed Polycaprolactone (PCL) flow diverting stent (FDS) with different lengths and diameters; (**b**) SEM images of the FDS surfaces and the width measurements; (**c**) Surface topography of the PCL FDS surfaces in 3D profilometer and (**d**) sub-micrometer scale wavy nature of the FDS surface topography.

**Figure 3:**
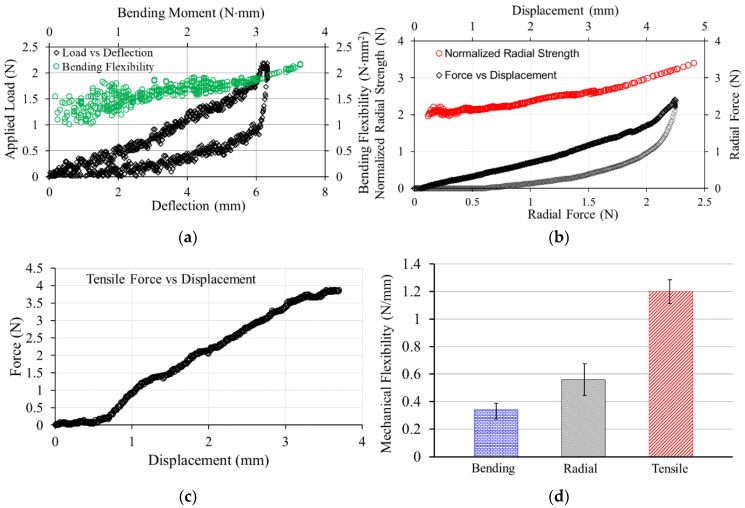
Mechanical strength and flexibility analysis of a Polycaprolactone (PCL) flow diverting stent (FDS): (**a**) three-point bending test; (**b**) flat plate radial compression test; (**c**) tensile force—displacement in longitudinal tension test and (**d**) average FDS flexibility in bending, radial and tensile experiment of FDS with ±1 standard deviation error bar.

Macroscopic views of developed PCL FDS, SEM image of the FDS and 3D profilometer mapping of the FDS surface are shown in Figure 2. SEM measurement shows that the average strut width was about 350 μm. The pore area ranged from 0.05 mm^2^ to 0.25 mm^2^. The average porosity of the developed PCL FDS was about 65% i.e., FDS surface coverage by the PCL was about 35%. The pore density was found to be about 0.87 pores/mm^2^. The surface of the FDS was generally very smooth. The 3D profilometer showed the sub-micrometer scale wavy topography of the FDS surfaces. The applied load vs. deflection curve with unloading nature is shown in Figure 3a for the 3-point bending test. The bending deflection quickly recovered with unloading as seen in Figure 3a. The bending flexibility of the FDS was also calculated and plotted in the same figure (Figure 3a) against bending moment. The radial force vs. displacement curve, along with the normalized radial strength in relation to the applied force and unloading nature, is shown in Figure 3b. The longitudinal force vs. displacement result is shown in Figure 3c. The bending deflection, radial compression, and longitudinal elongation were found to be 0.34 ± 0.03 N/mm, 0.56 ± 0.11 N/mm and 1.20 ± 0.19 N/mm, respectively, as seen in Figure 3d.

### 3.2. BaSO_4_ Coating for X-ray Image Analysis

**Figure 4:**
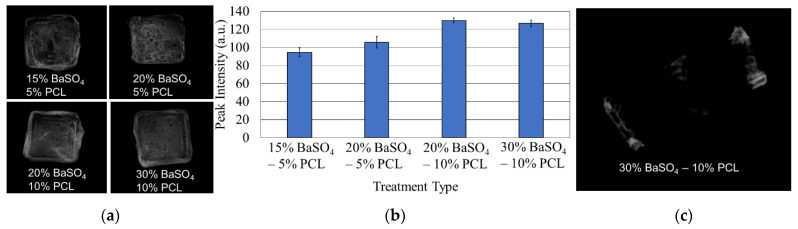
(**a**) X-ray images of flat PCL tiles coated with 15% BaSO_4_ and 5% PCL, 20% BaSO_4_ and 5% PCL, 20% BaSO_4_ and 10% PCL, and 30% BaSO_4_ and 10% PCL; (**b**) average peak intensity of coated PCL tiles with ± 1 standard error bars; and (**c**) PCL FDS ends coated with 30% BaSO_4_ and 10% PCL.

The X-ray images of the BaSO_4_ -PCL coated samples can be seen in Figure 4a. The average peak intensities ± 1 standard error of the mean of the 15% BaSO_4_: 5% PCL, 20% BaSO_4_: 5% PCL, 20% BaSO_4_: 10% PCL, and 30% BaSO_4_: 10% PCL coatings were found to be 94.6 ± 4.8 a.u. (arbitrary units), 105.7 ± 6.6 a.u., 129.9 ± 2.5 a.u., 126.8 ± 3.4 a.u., respectively. Figure 4b shows a graphical representation of this data. An X-ray image of a PCL FDS sample coated on the ends with the 30% BaSO_4_ and 10% PCL mixture is shown in Figure 4c.

**Figure 5:**
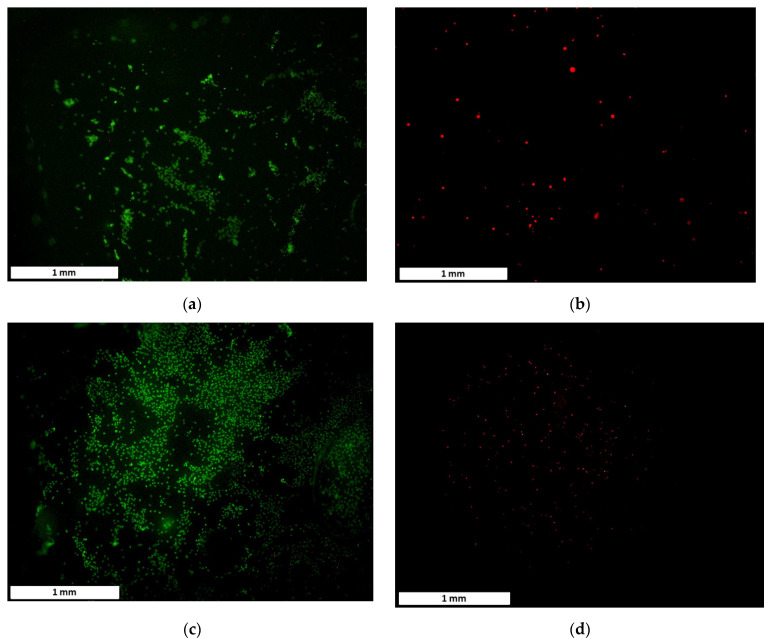
The effect of BaSO_4_ coating on the endothelial cell (HUVEC) functions—without BaSO_4_ coating (**a**) adhered cells on the PCL tile, (**b**) proliferated cells on the PCL tile; and with 30% BaSO_4_ and 10% PCL coating (**c**) adhered cells on the PCL tile and (**d**) proliferated cells on the PCL tile.

**Figure 6:**
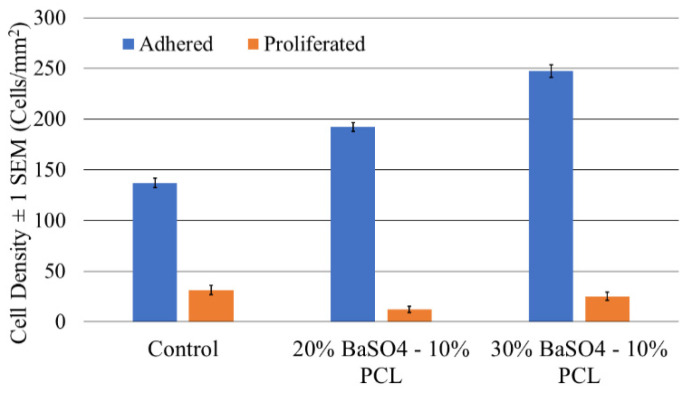
Average cell density ± 1 standard error bars of adhered and proliferated endothelial cells on PCL tiles with and without BaSO_4_ coating incubated for 24 h.

The results of the endothelial cell proliferation and adhesion responses to BaSO_4_ coating on the PCL tile surface are shown in Figure 5 and Figure 6. The average cell adhesion density ± standard error results for the NucSpot 470^®^ nuclear stain of the PCL control and coated tile samples was found to be 136.9 ± 4.7, 191.9 ± 4.4, and 247.2 ± 6.0 cells/mm^2^ for the control, 20% BaSO_4_: 10% PCL, and 30% BaSO_4_: 10% PCL coatings, respectively. The average cell density ± standard error results for the BaseClick™ 594-EdU cell proliferation stain of the control, the 20% BaSO_4_: 10% PCL, and the 30% BaSO_4_: 10% PCL coatings were found to be 31.4 ± 4.6, 12.1 ± 2.9, and 25.0 ± 4.0 cells/mm^2^, respectively.

### 3.3. HUVEC Cytotoxicity and NO Production Studies

**Figure 7:**
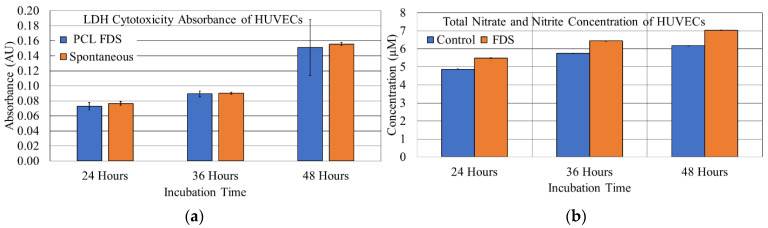
Quantification of (**a**) LDH release with LDH Cytotoxicity Assay with ± 1 standard error bars and (**b**) Nitrate/Nitrite Concentration with ± 1 standard error bars from HUVECs seeded with PCL FDS and well plate as the control for 24, 36, and 48 h of incubation period.

The LDH cytotoxicity assay absorbance results ± 1 standard error for the spontaneous control wells of the 24-, 36- and 48-h incubated samples were 0.077 ± 0.002 absorbance unit (AU), 0.090 ± 0.001 AU, 0.156 ± 0.002 AU, respectively. The absorbance results ± 1 standard error for the 24, 36, and 48-h incubated FDS samples were 0.073 ± 0.005 AU, 0.089 ± 0.001 AU, 0.151 ± 0.002 AU, respectively. These results are represented graphically in Figure 7a. The average concentration of total nitrate and nitrite ± 1 standard deviation in the control samples for the 24, 36, and 48-h incubation times were 4.872 ± 0.009 µM, 5.747 ± 0.006 µM, and 6.177 ± 0.004 µM, respectively. The average concentration of total nitrate and nitrite ± 1 standard deviation for the FDS treated samples were 5.495 ± 0.010 µM, 6.444 ± 0.002 µM, and 7.037 ± 0.002 µM, respectively. The addition of the PCL FDS samples to the cells resulted in a 12.8%, 12.1%, and 13.9% increase in the NO production of the 24-, 36-, and 48-h samples, respectively. A graphical representation of this data can be seen in Figure 7b.

### 3.4. Proliferation and Adhesion Analysis

**Figure 8:**
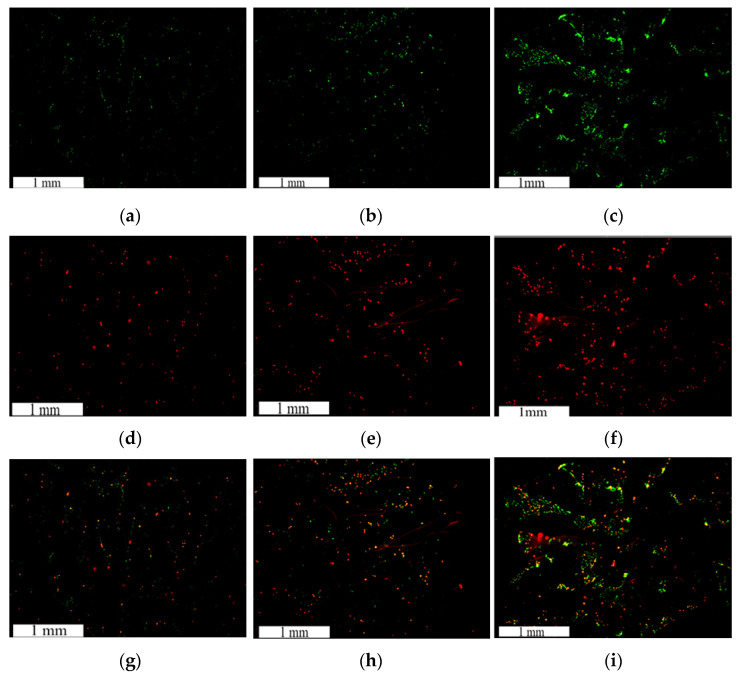
NucSpot 470^®^ cell adhesion Stain (Green-(**a**–**c)**), BaseClick™ 594-EdU cell proliferation stain (Red-(**d**–**f)**), and combined imaging (**g**–**i**) results for endothelial cells seeded on PCL FDS samples incubated for 24-h (**a**,**d**,**g**), 36-h (**b**,**e**,**h**), and 48-h (**c**,**f**,**i**).

**Figure 9:**
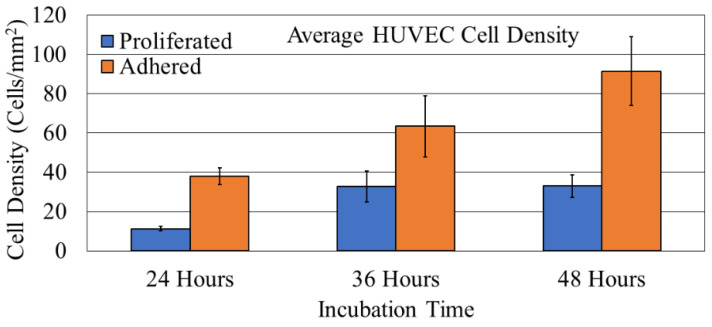
Average cell density ± 1 standard error bars of adhered and proliferated endothelial cells on Polycaprolactone (PCL) flow diverting stent (FDS) samples incubated for 24, 36, and 48 h.

The images obtained from microscopic fluorescent analysis of the PCL FDS cell proliferation and adhesion samples are shown in Figure 8. The reported cell density of the proliferation and adhesion stains was determined based on the FDS surface area. The average cell adhesion density ± standard error results for the NucSpot 470^®^ nuclear stain of the 24-, 36-, and 48-h samples were found to be 54.37 ± 5.97, 90.71 ± 22.20, and 130.71 ± 25.14 cells/mm^2^, respectively. The average cell density ± standard error results for the BaseClick™ 594-EdU cell proliferation stain of the 24-, 36-, and 48-h samples were found to be 16.11 ± 1.65, 46.83 ± 11.20, and 47.22 ± 8.14 cells/mm^2^, respectively. A graphical representation of this data is shown in Figure 9.

### 3.5. Cell Morphology

**Figure 10:**
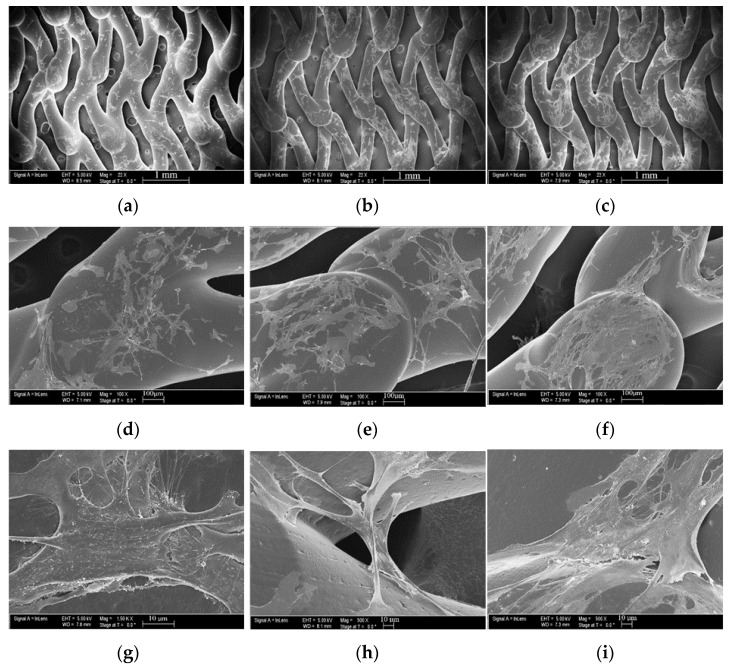
Cell morphology SEM imaging results for HUVECs seeded on PCL FDS samples incubated for 24-(**a**,**d**,**g**), 36-(**b**,**e**,**h**), and 48-h (**c**,**f**,**i**) with 1 mm (**a**–**c**), 100 µm (**d**–**f**), and 10 µm (**g**–**i**) scale bars.

The PCL FDS samples with seeded endothelial cells were imaged using a Zeiss Neon SEM under high magnification. The endothelial cell morphology SEM images of the 24-, 36-, and 48-h incubated samples can be seen in Figure 10. The cell morphology was analyzed and compared across incubation times. From Figure 10a–f it can be seen that over time the HUVECs begin to cover the surface of the FDS more fully. Figure 10g depicts a cell from a 24-h sample extending a filopodium to attach itself to the FDS surface while 10h shows cells from a 36-h sample elongating over the gap in the FDS structure. Finally, Figure 8i shows multiple cells flattening out over the FDS surface to form an endothelial monolayer over a 48-h sample.

## 4. Discussion

FDS surfaces act as a scaffold for developing endothelium and provide radial support to the blood vessel [46]. The SEM image of PCL FDS surfaces shows that the surface is smooth, while sub-micrometer wavy topography was found in the 3D profilometer. The smoother surface can be attributed to the single and thin layer printing strategies. Studies show that layer-by-layer printing is one reason for the surface roughness of the fused deposition modeling-based manufacturing of parts [47]. A smoother surface reduces the chance of vessel injuries during the deployment or repositioning of the FDS after deployment. The porosity and pore density of the developed FDS are comparable to the current laser-cut and braided neurovascular flow diverters [48]. The bending deflection rate increased with the applied load increase. Thus, bending flexibility increased with the bending moment. The bending flexibility of PCL FDS was higher than the coronary stent, as reported in [44]. The unloading curve of the bending deflection shows that PCL FDS can quickly recover from the bending deflection. The normalized radial strength increases with the applied radial forces. This means that resistance to radial deformation increases as the diameter of the FDS decreases with radial compression. The unloading curve of the radial compression shows nonlinear recovery as seen with typical coronary stent materials such as nitinol [49]. It also shows that the radial resistive force will be higher with higher deformation. It is notable that the developed FDS returns to its original shape and nearly to its initial diameter after being fully compressed and bent without compromising mechanical integrity. The longitudinal strength analysis shows the classical tensile force-tensile elongation curve nature. The overall bending deflection, radial compression, and longitudinal elongation per unit of applied force are comparable to the metallic balloon-expandable stent.

In order to benefit future practical applications of the PCL FDS, we explored an X-ray visible coating on the ends of the stent. During the deployment of FDS in vivo applications, a thin, X-ray visible coating on each end of the stent is desired for angiographic visualization during navigation. BaSO_4_ has been demonstrated as a stable, low toxic, and relatively less expensive radiopacifier for medical implants [50,51,52]. Based on the result of the individual X-ray intensity analyses and the convenience of the applicability as a very thin layer, the 30% BaSO_4_–10% PCL mixture worked best for the PCL FDS. While the 20% BaSO_4_–10% PCL mixture provided the most intense X-ray images, its viscosity made it extremely difficult to work with. The 2:1 ratio of BaSO_4_ to PCL created a very tacky, quickly drying, clumpy mixture that led to a thick, stringy coating. The thick coating compromised the bending flexibility and radial compressibility. For practical applications in vivo, a nice thin coating on the ends of the stent will be imperative for FDS function. We showed that BaSO_4_ coating improved cell adhesion on all of the coated samples. Proliferation on the 30% BaSO_4_ sample was not significantly different from control. The 20% BaSO_4_ sample had a slightly decreased proliferation density, although this is likely due to the increased number of adhered cells occupying space, resulting in contact inhibition. Our results also indicate that BaSO_4_ coating has no adverse effects on HUVEC adhesion and proliferation, and could likely improve the biocompatibility of the PCL FDS. These results are not only corroborated by previous studies [53], but also by years of clinical, in vivo use of BaSO_4_ as an X-ray contrast medium [54].

The LDH cytotoxicity assay results for the cells plated on the FDS samples were found to have no statistically significant difference from the cells plated in the spontaneous control wells. The nearly identical LDH levels of the FDS cells and the control cells indicate that the PCL FDS samples had no toxic effect on the HUVECs. This data corroborates previous studies that found PCL had no toxic effect on cells as well as enhanced antimicrobial properties [55]. NO production is an endothelial cell’s regulatory response to reduce the risk of atherosclerosis and thrombosis during stressful events within the endothelium [56]. Our NO production assay results are further indicative of the biocompatibility of PCL. The increase in NO production by the PCL FDS sample cells is supported by previous studies of NO production on PCL structures [57]. This result suggests that PCL would be capable of providing a healthy environment for endothelial cell NO production, which, in turn, would benefit the vascular wall healing process and reduce the risk of thrombosis and platelet aggregation.

We showed that endothelial cells not only adhere to the PCL FDS but also proliferate well on the surface. The marked increase in both adhered and proliferated cells between 24- and 36-h support this finding. When comparing the 36- and 48-h samples, there was still an increase in adhered cells; however, the number of proliferated cells stayed the same. This is not surprising given that the 36- and 48-h samples had the same amount of time with the fluorescent nucleoside. However, the 48-h samples had an extra twelve hours to allow the cells to fill the surface of the PCL FDS before the introduction of the nucleoside. Endothelial proliferation is known for being dependent upon surface attachment and is largely affected by contact inhibition [58]. As shown in Figure 8i, the cells grew to fill the space of the PCL FDS before we introduced the nucleoside, leading to a decreased proliferation rate during the nucleoside incubation period. Overall, this result supports the theory that PCL would be a biocompatible alternative to current metal-based FDSs. The HUVECs on the PCL FDS surface demonstrate an elongated, flattened cell morphology. The image of a 24-h incubated sample in Figure 10g shows a cell extending its filopodium to attach to and flatten along the FDS surface. Figure 10h shows a cell from a 36-h incubated sample bridging the gap of a corner of the FDS pore, demonstrating the potential for pore coverage. An image from a 48-h incubated sample shown in Figure 10i captures the monolayer coverage of the FDS surface, which supports the biocompatibility of the PCL. These results demonstrate the likelihood that endothelial cells will form a monolayer covering the FDS surfaces. In summary, the morphology results further indicate that the PCL FDS structure supports endothelial cell adhesion, proliferation, and monolayer formation. Nonetheless, our study has limitations such as the compression test was done with a flat plate model where uniform and non-uniform radial compression can provide better insight of the FDS design, detailed degradation analysis with decompositions chemistry, and thrombogenicity studies. Finally, in vivo efficacy studies with animal models will be essential to determine whether biodegradable FDS is clinically safe, efficient and functional for brain aneurysm treatment. Therefore, this study is not claiming that the reported PCL flow diverter is ready for clinical application. Instead, we hope that this study will contribute to developing functional and clinically sound biodegradable flow diverting stents. Our future studies will address the above limitations of the non-braided biodegradable flow diverting stents.

## 5. Conclusions

This research presents a novel biodegradable and non-braided PCL FDS and fabrication process. SEM and 3D profilometer characterization showed that surfaces demonstrate sub-micrometer scale wavy topography. The bending and radial flexibility, radial and longitudinal strength were comparable to the metallic balloon-expandable coronary stents. The FDS returned to its normal shape and nearly to its initial diameter after full compression and bending. BaSO_4_ coating was stable, non-toxic and provided good clarity under X-ray imaging. The cytotoxicity results with LDH release by the HUVECs with PCL FDS showed no toxicity, while NO production also showed positive results. HUVEC adhesion and proliferation on the PCL FDS showed higher density over time. The cell morphology studies indicated the formation of an endothelial monolayer on the FDS surfaces. The promising results of this study are a step forward to develop fully biodegradable flow diverting stents for endovascular treatment of aneurysms.

## Data Availability

Not applicable.

## References

[1] Shin D.S., Carroll C.P., Elghareeb M., Hoh B.L., Kim B.T. (2020). The evolution of flow-diverting stents for cerebral aneurysms; historical review, modern application, complications, and future direction. J. Korean Neurosurg. Soc..

[2] Chong W., Zhang Y., Qian Y., Lai L., Parker G., Mitchell K. (2014). Computational hemodynamics analysis of intracranial aneurysms treated with flow diverters: Correlation with clinical outcomes. Am. J. Neuroradiol..

[3] Arrese I., Sarabia R., Pintado R., Delgado-Rodriguez M. (2013). Flow-diverter devices for intracranial aneurysms: Systematic review and meta-analysis. Neurosurgery.

[4] Briganti F., Leone G., Marseglia M., Mariniello G., Caranci F., Brunetti A., Maiuri F. (2015). Endovascular treatment of cerebral aneurysms using flow-diverter devices: A systematic review. Neuroradiol. J..

[5] Brinjikji W., Murad M.H., Lanzino G., Cloft H.J., Kallmes D.F. (2013). Endovascular treatment of intracranial aneurysms with flow diverters: A meta-analysis. Stroke.

[6] Korkmazer B., Kocak B., Islak C., Kocer N., Kizilkilic O. (2019). Long-term results of flow diversion in the treatment of intracranial aneurysms: A retrospective data analysis of a single center. Acta Neurochir..

[7] Hofma S.H., Whelan D.M.C., Van Beusekom H.M.M., Verdouw P.D., Van der Giessen W.J. (1998). Increasing arterial wall injury after long-term implantation of two types of stent in a porcine coronary model. Eur. Heart J..

[8] Peschillo S., Caporlingua A., Resta M.C., Peluso J.P.P., Burdi N., Sourour N., Diana F., Guidetti G., Clarençon F., Bloemsma G.C. (2017). Endovascular treatment of large and giant carotid aneurysms with flow-diverter stents alone or in combination with coils: A multicenter experience and long-term follow-up. Oper. Neurosurg..

[9] Petr O., Brinjikji W., Cloft H., Kallmes D.F., Lanzino G. (2016). Current trends and results of endovascular treatment of unruptured intracranial aneurysms at a single institution in the flow-diverter era. Am. J. Neuroradiol..

[10] Brancati M.F., Burzotta F., Trani C., Leonzi O., Cuccia C., Crea F. (2017). Coronary stents and vascular response to implantation: Literature review. Pragmat. Obs. Res..

[11] Crimi G., Gritti V., Galiffa V.A., Scotti V., Leonardi S., Ferrario M., Ferlini M., De Ferrari G.M., Oltrona Visconti L., Klersy C. (2018). Drug eluting stents are superior to bare metal stents to reduce clinical outcome and stent-related complications in CKD patients, a systematic review, meta-analysis and network meta-analysis. J. Interv. Cardiol..

[12] Kuramitsu S., Jinnouchi H., Shinozaki T., Hiromasa T., Matsumura Y., Yamaji Y., Miura M., Matsuda H., Masuda H., Domei T. (2017). Incidence and long-term clinical impact of late-acquired stent fracture after sirolimus-eluting stent implantation in narrowed coronary arteries. Am. J. Cardiol..

[13] Egbuche O., Mezue K.N., Nwokike S.I., Abe T., Olanipekun T., Onuorah I., Tharpe C. (2021). Left main stenting with stent dislodgement and entrapment in the common femoral artery: A successful transcatheter stent retrieval. Am. J. Cardiovasc. Dis..

[14] Hu T., Yang C., Lin S., Yu Q., Wang G. (2018). Biodegradable stents for coronary artery disease treatment: Recent advances and future perspectives. Mater. Sci. Eng. C.

[15] Onuma Y., Piazza N., Ormiston J.A., Serruys P.W. (2009). Everolimus-eluting bioabsorbable Stent-Abbot vascular programme. EuroIntervention.

[16] Tenekecioğlu E., Bourantas C., AbdelGhani M., Zeng Y., Silva R.C., Tateishi H., Sotomi Y., Onuma Y., Yılmaz M., Serruys P.W. (2016). From drug eluting stents to bioresorbable scaffolds; to new horizons in PCI. Expert Rev. Med. Devices.

[17] Sotomi Y., Onuma Y., Collet C., Tenekecioglu E., Virmani R., Kleiman N.S., Serruys P.W. (2017). Bioresorbable scaffold: The emerging reality and future directions. Circ. Res..

[18] Waksman R. (2006). Biodegradable stents: They do their job and disappear. J. Invasive Cardiol..

[19] Simard T., Hibbert B., Ramirez F.D., Froeschl M., Chen Y.X., O’Brien E.R. (2014). The evolution of coronary stents: A brief review. Can. J. Cardiol..

[20] Hytönen J.P., Taavitsainen J., Tarvainen S., Ylä-Herttuala S. (2018). Biodegradable coronary scaffolds: Their future and clinical and technological challenges. Cardiovasc. Res..

[21] Pauck R.G., Reddy B.D. (2015). Computational analysis of the radial mechanical performance of PLLA coronary artery stents. Med. Eng. Phys..

[22] Qiu T., Zhao L. (2018). Research into biodegradable polymeric stents: A review of experimental and modelling work. Vessel. Plus.

[23] Ormiston J.A., Serruys P.W. (2009). Bioabsorbable coronary stents. Circ. Cardiovasc. Interv..

[24] Schmidt W., Behrens P., Brandt-Wunderlich C., Siewert S., Grabow N., Schmitz K.P. (2016). In vitro performance investigation of bioresorbable scaffolds–standard tests for vascular stents and beyond. Cardiovasc. Revasculariz. Med..

[25] Welch T.R., Eberhart R.C., Reisch J., Chuong C.J. (2014). Influence of thermal annealing on the mechanical properties of PLLA coiled stents. Cardiovasc. Eng. Technol..

[26] Yang G., Xie H., Huang Y., Lv Y., Zhang M., Shang Y., Zhou J., Wang L., Wang J.-Y., Chen F. (2017). Immersed multilayer biodegradable ureteral stent with reformed biodegradation: An in vitro experiment. J. Biomater. Appl..

[27] Qiu T., He R., Abunassar C., Hossainy S., Zhao L.G. (2018). Effect of two-year degradation on mechanical interaction between a bioresorbable scaffold and blood vessel. J. Mech. Behav. Biomed. Mater..

[28] Liu R., Xu S., Luo X., Liu Z. (2020). Theoretical and numerical analysis of mechanical behaviors of a metamaterial-based shape memory polymer stent. Polymers.

[29] Hutmacher D.W., Schantz T., Zein I., Ng K.W., Teoh S.H., Tan K.C. (2001). Mechanical properties and cell cultural response of polycaprolactone scaffolds designed and fabricated via fused deposition modeling. J. Biomed. Mater. Res. Off. J. Soc. Biomater. Jpn. Soc. Biomater. Aust. Soc. Biomater. Korean Soc. Biomater..

[30] Semba T., Kitagawa K., Ishiaku U.S., Hamada H. (2006). The effect of crosslinking on the mechanical properties of polylactic acid/polycaprolactone blends. J. Appl. Polym. Sci..

[31] Woodruff M.A., Hutmacher D.W. (2010). The return of a forgotten polymer—Polycaprolactone in the 21st century. Prog. Polym. Sci..

[32] Ang H.Y., Bulluck H., Wong P., Venkatraman S.S., Huang Y., Foin N. (2017). Foin Bioresorbable stents: Current and upcoming bioresorbable technologies. Int. J. Cardiol..

[33] Estellés J.M., Vidaurre A., Duenas J.M.M., Cortázar I.C. (2008). Physical characterization of polycaprolactone scaffolds. J. Mat. Sci. Mat. Med..

[34] Bastioli C. (2020). Handbook of Biodegradable Polymers.

[35] Khandaker M., Riahinezhad S., Jamadagni H.G., Morris T.L., Coles A.V., Vaughan M.B. (2017). Use of polycaprolactone electrospun nanofibers as a coating for poly(methyl methacrylate) bone cement. Nanomaterials.

[36] Park S.H., Park S.A., Kang Y.G., Shin J.W., Park Y.S., Gu S.R., Wu Y.R., Wei J., Shin J.W. (2017). PCL/β-TCP composite scaffolds exhibit positive osteogenic differentiation with mechanical stimulation. Tissue Eng. Regen. Med..

[37] East B., Plencner M., Kralovic M., Rampichova M., Sovkova V., Vocetkova K., Otahal M., Tonar Z., Kolinko Y., Amler E. (2018). A polypropylene mesh modified with poly-ε-caprolactone nanofibers in hernia repair: Large animal experiment. Int. J. Nanomed..

[38] Oh S.H., Park I.K., Kim J.M., Lee J.H. (2007). In vitro and in vivo characteristics of PCL scaffolds with pore size gradient fabricated by a centrifugation method. Biomaterials.

[39] Sayyar S., Murray E., Thompson B.C., Gambhir S., Officer D.L., Wallace G.G. (2013). Wallace Covalently linked biocompatible graphene/polycaprolactone composites for tissue engineering. Carbon.

[40] Zhu Y., Gao C., Liu X., Shen J. (2002). Surface modification of polycaprolactone membrane via aminolysis and biomacromolecule immobilization for promoting cytocompatibility of human endothelial cells. Biomacromolecules.

[41] Guerra A.J., Ciurana J. (2018). Design 3D-printed bioabsordable polycaprolactone stent: The effect of process parameters on its physical features. Mater. Des..

[42] Qiu T., Jiang W., Yan P., Jiao L., Wang X. (2020). Development of 3D-printed sulfated chitosan modified bioresorbable stents for coronary artery disease. Front. Bioeng. Biotechnol..

[43] Coombes A.G.A., Rizzi S.C., Williamson M., Barralet J.E., Downes S., Wallace W.A. (2004). Wallace Precipitation casting of polycaprolactone for applications in tissue engineering and drug delivery. Biomaterials.

[44] Yokoo T., Shimizu I., Wada A., Takaki A., Okada S., Hatakeyama M., Yamashita S. (2014). Development of Test Methods for Mechanical Property Evaluation of Balloon-Expandable CoCr Alloy Stent. J. Jpn. Soc. Exp. Mech..

[45] McGrath D., O’Brien B., Bruzzi M., Kelly N., Clauser J., Steinseifer U., McHugh P. (2016). Evaluation of cover effects on bare stent mechanical response. J. Mech. Behav. Biomed. Mater..

[46] Ravindran K., Casabella A.M., Cebral J., Brinjikji W., Kallmes D.F., Kadirvel R. (2020). Mechanism of action and biology of flow diverters in the treatment of intracranial aneurysms. Neurosurgery.

[47] Taufik M., Jain P.K. (2020). Part surface quality improvement studies in fused deposition modelling process: A review. Aust. J. Mech. Eng..

[48] Dholakia R., Sadasivan C., Fiorella D.J., Woo H.H., Lieber B.B. (2017). Hemodynamics of flow diverters. J. Biomech. Eng..

[49] Stöckel D. (1998). Nitinol-A material with unusual properties. Endovasc. Update.

[50] Boyer C.J., Boktor M., Samant H., White L.A., Wang Y., Ballard D.H., Huebert R.C., Woerner J.E., Ghali G.E., Alexander J.S. (2019). 3D printing for bio-synthetic biliary stents. Bioengineering.

[51] Lämsä T., Jin H., Mikkonen J., Laukkarinen J., Sand J., Nordback I. (2006). Biocompatibility of a New Bioabsorbable Radiopaque Stent Material (BaSO4 Containing Poly-L, D-Lactide) in the Rat Pancreas. Pancreatology.

[52] Ang H.Y., Toong D., Chow W.S., Seisilya W., Wu W., Wong P., Venkatraman S.S., Foin N., Huang Y. (2018). Radiopaque fully degradable nanocomposites for coronary stents. Sci. Rep..

[53] Murray P.E., Lumley P.J., Ross H.F., Smith A.J. (2000). Tooth slice organ culture for cytotoxicity assessment of dental materials. Biomaterials.

[54] Garrett P.R., Meshkov S.L., Perlmutter G.S. (1984). Oral contrast agents in CT of the abdomen. Radiology.

[55] Uscátegui Y.L., Arévalo F.R., Díaz L.E., Cobo M.I., Valero M.F. (2016). Microbial degradation, cytotoxicity and antibacterial activity of polyurethanes based on modified castor oil and polycaprolactone. J. Biomat. Sci. Polym. Ed..

[56] Forstermann U., Munzel T. (2006). Endothelial nitric oxide synthase in vascular disease: From marvel to menace. Circulation.

[57] Serrano M.C., Pagani R., Vallet-Regí M., Peña J., Comas J.V., Portolés M.T. (2009). Nitric oxide production by endothelial cells derived from blood progenitors cultured on NaOH-treated polycaprolactone films: A biofunctionality study. Acta Biomater..

[58] Gérard C., Goldbeter A. (2014). The balance between cell cycle arrest and cell proliferation: Control by the extracellular matrix and by contact inhibition. Interface Focus.

